# Short Disordered Epitope of CRTAM Ig-Like V Domain as a Potential Target for Blocking Antibodies

**DOI:** 10.3390/ijms21228798

**Published:** 2020-11-20

**Authors:** Julio Angel Vázquez-Martínez, Miguel Angel Gómez-Lim, Edgar Morales-Ríos, Jorge Alberto Gonzalez-y-Merchand, Vianney Ortiz-Navarrete

**Affiliations:** 1 Departamento de Microbiología, Escuela Nacional de Ciencias Biológicas, Instituto Politécnico Nacional, 11340 Ciudad de Mexico, Mexico; jvazquezm1408@alumno.ipn.mx (J.A.V.-M.); jgonzal1212@yahoo.com.mx (J.A.G.-y.-M.); 2 Departamento de Biomedicina Molecular, Centro de Investigación y Estudios Avanzados del Instituto Politécnico Nacional (CINVESTAV), 07360 Ciudad de Mexico, Mexico; 3 Departamento de Ingeniería Genética, Centro de Investigación y Estudios Avanzados del Instituto Politécnico Nacional (CINVESTAV), 36821 Irapuato, Guanajuato, Mexico; miguel.gomez@cinvestav.mx; 4 Departamento de Bioquímica, Centro de Investigación y Estudios Avanzados del Instituto Politécnico Nacional (CINVESTAV), 07360 Ciudad de Mexico, Mexico; edgar.morales@cinvestav.mx

**Keywords:** Intrinsically Disordered Regions, short-disordered epitopes, CRTAM, Necl2, VLPs

## Abstract

Class-I Restricted T Cell-Associated Molecule (CRTAM) is a protein that is expressed after T cell activation. The interaction of CRTAM with its ligand, nectin-like 2 (Necl2), is required for the efficient production of IL-17, IL-22, and IFNγ by murine CD4 T cells, and it plays a role in optimal CD8 T and NK cell cytotoxicity. CRTAM promotes the pro-inflammatory cytokine profile; therefore, it may take part in the immunopathology of autoimmune diseases such as diabetes type 1 or colitis. Thus, antibodies that block the interaction between CRTAM and Necl2 would be useful for controlling the production of these inflammatory cytokines. In this work, using bioinformatics predictions, we identified three short disordered epitopes (sDE1-3) that are located in the Ig-like domains of murine CRTAM and are conserved in mammalian species. We performed a structural analysis by molecular dynamics simulations of sDE1 (QHPALKSSKY, Ig-like V), sDE2 (QRNGEKSVVK, Ig-like C1), and sDE3 (CSTERSKKPPPQI, Ig-like C1). sDE1, which is located within a loop of the contact interface of the heterotypic interaction with Nectl2, undergoes an order–disorder transition. On the contrary, even though sDE2 and sDE3 are flexible and also located within loops, they do not undergo order–disorder transitions. We evaluated the immunogenicity of sDE1 and sDE3 through the expression of these epitopes in chimeric L1 virus-like particles. We confirmed that sDE1 induces polyclonal antibodies that recognize the native folding of CRTAM expressed in activated murine CD4 T cells. In contrast, sDE3 induces polyclonal antibodies that recognize the recombinant protein hCRTAM-Fc, but not the native CRTAM. Thus, in this study, an exposed disordered epitope in the Ig-like V domain of CRTAM was identified as a potential site for therapeutic antibodies.

## 1. Introduction

Intrinsically disordered proteins (IDPs) are molecules that undergo conformational transition; they do not adopt a unique folded structure in their native state because they are highly dynamic and rich in polar residues [1,2,3,4]. This characteristic allows IDPs to act as hub or scaffold molecules in protein–protein interaction networks with different ligands (binding promiscuity) and participate in the modulation of many cellular processes, such as cell cycle regulation [5], signaling pathways, transcription, and translation [6,7,8]. These IDPs are subject to post-translational modifications (PTMs) such as phosphorylation, which can promote or disrupt protein–protein interactions, signal pathways, molecular traffic, and the half-life [9] and degradation of proteins [8,10,11,12]. Some proteins have well-conformed domains and disordered segments in their structures that are similar to disordered proteins; these sequences are known as intrinsically disordered regions (IDRs) [13]. Typical characteristics of these IDRs include solvent accessibility and transitional order–disorder states in the absence of ligands. Thus, they have critical roles as short linear binding motifs (SLiMs) [14,15] or molecular recognition features (MoRFs) [16]. Despite the high molecular disorder of pathogen or endogenous mammalian proteins, the hydrophilicity of exposed residues and the lengths of IDRs play roles in stimulating the immune system. There are several antigens under development as vaccines that have been predicted to be disordered proteins, such as the circumsporozoite protein and merozoite surface 2 of *Plasmodium falciparum*, the KMP-11 protein of *Leishmania amazonensi*, the preS antigen of hepatitis B, glycoprotein G of the herpes simplex virus, and the Neisserial heparin binding antigen of *Neisseria meningitidis* serogroup B [17]. In addition, IDRs can be highly immunogenic linear or conformational B-cell epitopes. IDRs in immunogenic linear B-cell epitopes have been identified for the neuraminidase and hemagglutinin proteins of the avian influenza virus H5N1 and H9N2, and they were suggested as candidates to produce a vaccine for human and poultry use [18]. Furthermore, antibodies with high affinity and specificity against IDR epitopes of α-synuclein, the Aβ peptide, and the islet amyloid polypeptide have been designed. Notably, these proteins are involved in Parkinson’s, Alzheimer’s, and type II diabetes, respectively.

On the other hand, CRTAM (Class-I Restricted T Cell-Associated Molecule) protein, also named CD355, belongs to the immunoglobulin (Ig) superfamily of proteins. CRTAM is a cell surface transmembrane protein that contains one Ig-like variable domain (Ig-V), one constant Ig-like domain (Ig-C), a stalk, a transmembrane region, and a cytoplasmic tail that carries one class-I PDZ (ESIV) binding motif that binds to the PDZ domains of scaffold proteins [19,20]. Bioinformatics analysis has classified it as a member of the nectin-like family [21]. CRTAM is expressed on the surface of in vitro activated iNKT, NK, and CD8 T cells and a subset of CD4 T cells in both humans and mice [19,20,21,22,23,24,25]. Activated mouse CRTAM^+^ CD4 T cells produce IFN-γ and IL-17/IL-22 [20]. Recently, it was demonstrated that IFN-γ produced by CRTAM^+^ CD4 T cells plays a role in the inflammatory process observed in the DSS-induced colitis mouse model [26]. CRTAM is detected at the cell membrane of CD4 and CD8 T cells, neutrophils, and basophils from patients with asthma, suggesting that these cells are involved in the immunopathology of this disease [27]. Moreover, after antigen-specific stimulation, a clear association between CRTAM expression and IFN-γ production in iNKT cells from healthy subjects and type 1 diabetes patients has been observed [25]. These findings indicate that CRTAM may also play a role in triggering the production of IFN-γ in human iNKT cells, and they strengthen the hypothesis that CRTAM-expressing cells might play a pathogenic role in inflammatory diseases through IFN-γ production [25]. 

CRTAM must interact with its ligand, nectin-like 2 (Necl2), before it can mediate IFN-γ production in activated NK, iNKT, CD8, and CD4 T cells [20,23,24,25,28]. Since IDRs can be binding sites for protein–protein interactions and can also be B-cell epitopes, we focused on identifying exposed epitopes with short IDRs in the extracellular region of CRTAM to generate antibodies for future therapeutic applications in treating inflammatory diseases.

## 2. Results and Discussion

### 2.1. CRTAM Protein Has Short Disordered Epitopes That Are Conserved in Mammals

**Figure 1:**
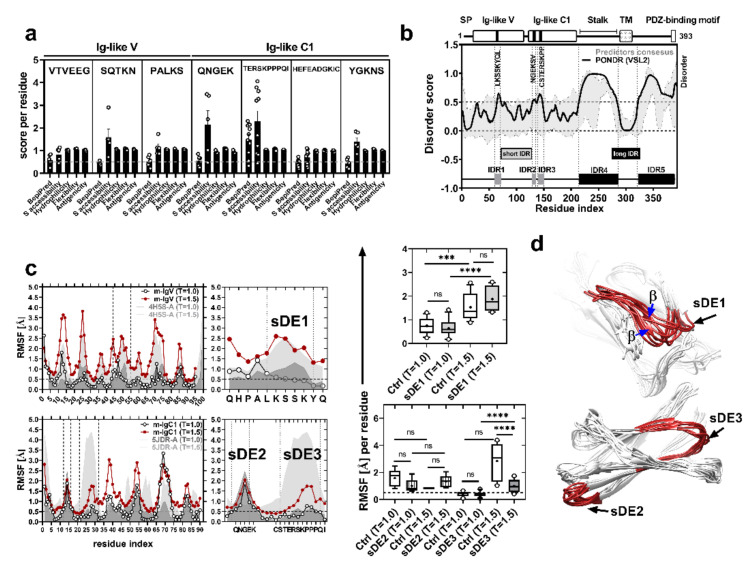
Short disordered epitopes of CRTAM Ig-like domains. (**a**) Linear B-cell epitope predictions by different algorithms (threshold = 0.5, 10% F.P). Each point represents the score for each residue of the epitope sequence. (**b**) Prediction of the intrinsically disordered region of the murine CRTAM sequence. The top panel shows the scheme of the CRTAM protein: Ig-like variable and constant domains, stalk, transmembrane (TM), cytoplasmic region, and PDZ-binding motif ESIV. The black lines in the Ig-like domains represent the locations of short disordered epitopes (sDEs); the predictor consensus is shaded in gray (standards errors from algorithms: IUPRED2A, PrDOS, DISOPRED3, and DisEMBL), and PONDR-VSL2 is indicated by the black line. The groups of residues (peaks) with scores of ≥0.5 are defined as short (gray boxes) or long (black boxes) intrinsically disordered regions (IDR1-5). (**c**) Behavior of the amino acid fluctuations according to CABS-flex coarse-grained molecular dynamics. The plot represents the RMSF of the control structure (4H5S-A) or murine CRTAM models (m-IgV or m-IgC1) in two conditions (T = 1.0 and T = 1.5). The RMSF averages of sDE1-3 are shown in the box and whisker (min to max) plots and were evaluated by one-way ANOVA (*** *p* < 0.001, **** *p* < 0.0001, ns = not significant). (**d**) Structures are representative of 10 models using CABS-flex MD at T = 1.5 from Ig-like V or C1 domains: each sDE is shown in red, and ambiguous structures are indicated with blue arrows.

In order to identify potential epitopes in the sequence of CRTAM Ig-like domains, we used tools for the prediction of linear epitopes by determining their solvent accessibility (sa), hydrophilicity (hp), flexibility (fl), and antigenicity (ag). A threshold of 0.2 was used for BepiPred prediction (sensitivity = 0.56 and specificity = 0.68). The results show seven linear epitopes in Ig-like domains: ^24^VTVEEG^29^ (sa = 1.145 ± 0.28, hp = 1.048 ± 0.02, fl = 1.026 ± 0.04, ag = 1.048 ± 0.02), ^39^SQTKN^43^ (sa = 2.158 ± 1.37, hp = 0.9452 ± 0.06, fl = 1.095 ± 0.02, ag = 0.9452 ± 0.06), ^62^PALKS^66^ (sa = 2.298 ± 1.42, hp = 1.004 ± 0.03, fl = 1.057 ± 0.03, ag = 1.004±0.03), ^129^QNGEK^133^ (sa = 0.8205 ± 0.28, hp = 1.014 ± 0.02, fl = 1.035 ± 0.04, ag = 1.014 ± 0.02), ^141^TERSKPPPQI^150^ (sa = 1.587 ± 0.80, hp = 1.021 ± 0.04, fl = 1.071 ± 0.03, ag = 1.021 ± 0.04), ^166^HEFEADGKIC^175^ (sa = 0.6926 ± 0.32, hp = 0.9802 ± 0.03, fl = 0.9981 ± 0.04, ag = 0.9802 ± 0.03), and ^138^YGKNS^149^ (sa = 1.386 ± 0.43, hp = 0.9768 ± 0.02, fl = 1.046 ± 0.04, 0.9768 ± 0.02). We also found four long epitopes (24–35 residues): two localized in the stalk and two in the cytoplasmic tail of CRTAM (Figure 1a and Figure S1a and Table S5). The residues of the epitope from murine CRTAM ^62^PALKS^66^ are similar to the sequence ^46^YPALK^50^ of human CRTAMv (PDB: 4H5S) in part of the contact interface of the heterotypic interaction with its ligand, nectin-like 2 (Necl2) [29]. This interaction promotes the efficient production of interferon gamma (IFN-γ) in CD8 cytotoxic T cells, NK cells, and NKT cells [19,25,29,30], which suggests that it is a promising blocking site. 

Evidence has shown that intrinsically disordered regions (IDRs), such as epitopes (disordered epitopes), are more enriched in linear B-cell epitopes, putative disordered binding sites, and disorder–order transitioning T cell epitopes [17,31,32]. Moreover, disordered epitopes can induce an adaptive immune response and efficient protective antibody production against pathogen surface proteins [18,33,34,35] due to the solvent exposure and entropic cost efficiency of the paratope–disordered epitope complex [36]. On the basis of these features, we investigated whether CRTAM epitopes are disordered epitopes. First, we analyzed the primary sequence of CRTAM using different disorder predictors. We found three short intrinsically disordered regions (IDRs): IDR1 (^65^LKSSKYQL^71^), which is located in the Ig-like V domain, and IDR2 (^130^NGEKSV^135^) and IDR3 (^139^CSTERSKPP^147^), both located in the Ig-like C domain. We also found two long intrinsically disordered regions: IDR4 (residues 215–283), corresponding to the stalk region, and IDR5 (residues 323–393), located in the cytoplasmic region (Figure 1b and Table S6). The results show that these disordered regions correspond to three epitopes from Ig-like domains: PA**LKSSKYQL**, Q**NGEKSV**, and **CSTERSKPP**PQI (hereafter, we refer to these epitopes as sDE1, sDE2, and sDE3), suggesting that they are disordered epitopes and potential antigenic regions for antibody production. Furthermore, the disorder score of the stalk region (IDR4; epitopes 8–9, residues 216–250 and 252–285, respectively) is consistent with its antigenicity potential. In a previous work, a linear antigenic peptide (^257^DKEEKE^262^) from the stalk region of CRTAM (here referred to as IDR4) was able to induce antibodies that recognized CRTAM expressed in MDCK epithelial cells and rat kidney cells [37]. These data suggest that disordered peptides are efficient in inducing a specific immune response, as reflected by their use in vaccine development [17,38,39]. 

Molecular disorder in the cytoplasmic tail has been described in 41% of human transmembrane proteins and receptors [40,41,42]. These disordered regions are critical binding sites [43] that are regulated by their ligands, post-translational modification [44], or autoinhibition [45]. Here, we found a high disorder score for the CRTAM cytoplasmic tail (IDR5; epitopes 10-11, residues 324–357 and 368–391, respectively), which correlates with the presence of the PDZ-binding motif (ESIV) in the C-terminal region of CRTAM [21]. This motif regulates and maintains the polarization of TCR, CD44, and Talin after T cell activation through its interaction with Scrib [20]. These data suggest that the predicted long IDRs of CRTAM have potential binding sites for protein–protein interactions due to their molecular disorder.

We hypothesized that Ig-like sDEs of CRTAM might be conserved among several species since they are important sites of interaction. To validate our assumption, we performed a multiple alignment of CRTAM sequences in the UniProtKB mammalian database. As expected, we observed that sDE1-3 of CRTAM, as well as the long IDR4-5 (Figure S2), are conserved in 55 mammalian species, including human, chimpanzee, gorilla, cat, and golden hamster (Figure S2 and Table S6). The data show that conserved short disordered epitopes 1–3 are potential candidates for antibody production. 

### 2.2. sDE1 Has a Flexible Loop Structure

Disordered regions have been described as protein segments that lack structures such as alpha helices or beta-sheets. They generally adopt a loop conformation, but when they interact with their ligand, they can shift to different conformational states [46]. In order to evaluate the folding of sDE from Ig-like domains, we generated structures of murine CRTAM based on the crystallographic structures of the human CRTAM Ig-like V domain (PDB: 4H5S-A). Because there is no crystallographic structure of the Ig-C domain of CRTAM, we searched for a protein that could be used for homology modeling. The structures of both PD-L1 and CRTAM have two extracellular Ig-like domains [23,47]; the Ig-like C2 of PD-L1 has 96 similar and 64 identical residue positions to those of the Ig-like C domain of CRTAM, according to the software used for modeling 3D structures. We found that the Ig-C domain of PD-L1 was the best template for generating the 3D structure of the Ig-C domain of CRTAM. Thus, we used the structure of PD1-L1 (PDB: 5JDR-A) [48]. The three-dimensional models of CRTAM Ig-like domains were refined and subsequently validated in a Ramachandran plot (Figure S3), and the models show that sDE1-3 have unstructured loops (Figure S4), which agrees with the disordered region prediction described above. To support the presence of short disordered epitopes, we performed coarse-grained molecular dynamics simulations of Ig-like models and control crystal structures (PDB: 4H5S and 5JDR). Thus, CABS-flex was used to evaluate the flexibility of these loops in two different conditions: crystallization conditions (T = 1) and with an increased temperature to increase the energy of the system (T = 1.5) based on the Boltzmann constant. The behavior of the residue fluctuations (RMSF) demonstrates that sDE1 from human Ig-like V (4H5S-A) or murine Ig-like V (m-IgV) has low flexibility (0.7427 and 0.6389 Å, respectively) at T = 1.0 (277 K and pH = 8.0). However, the flexibility increases in both structures (1.525 and 1.877 Å, respectively) at T = 1.5 (Figure 1c, top panel). On the other hand, at T = 1.0 (295 K), sDE2 from Ig-like C1 (m-IgC1) or the control model (5JDR-A) has moderate flexibility (1.527 and 0.9956 Å, respectively). Moreover, this characteristic does not change at T = 1.5 (0.8268 and 1.325 Å, respectively) (Figure 1c, bottom panel). In contrast, at T = 1.0, sDE3 from Ig-like C1 or the control model (5JDR-A) is in a rigid state (0.3658 and 0.4071 Å, respectively), and it remains rigid at T = 1.5 for sDE3 (0.9917 Å), but becomes more flexible in the control model (2.845 Å) (Figure 1c, bottom panel). These data are consistent with the conformational possibilities of the top ten 3D models obtained from the CABS-flex simulation (2000 models). Hence, we observed the formation of two beta-sheets in the sDE1 loop (Figure 1d, top panel), which suggests that heterogeneous folding or a disorder–order transition occurs in this zone. In contrast, we observed low flexibility and a lack of a disorder–order transition in the sDE2 and sDE3 loops (Figure 1d, bottom panel). We did not observe differences in the average relative solvent-accessible surface area (rSASA) of sDE in the control and query models (Figure S5a). According to IDR classification, we concluded that sDE1 is a short linear motif that is involved in ligand interaction [46] because it is highly flexible and has an ambiguous conformation. However, this short linear motif adopts a stable conformation in the crystallographic structure; the discrepancy with our model is likely because the conditions used to crystalize the Ig-V domain of CRTAM do not reflect the conditions of the native structure. Similarly, crystallographic structures represent a picture of the highly structured three-dimensional conformation, as reported previously [49,50]. In contrast, although the rSASA averages of sDE1-3 are similar, sDE2 and sDE3 have constant or low flexibility without structural divergence, which could impact their energy efficiency in the paratope–epitope complex [36]. Given these results, we experimentally evaluated the immunogenicity of two epitopes, one with high disorder (sDE1) and the other with low flexibility (sDE3), with the same exposure to the solvent.

### 2.3. Virus-Like Particles as Carriers of Short Disordered Epitopes

**Figure 2:**
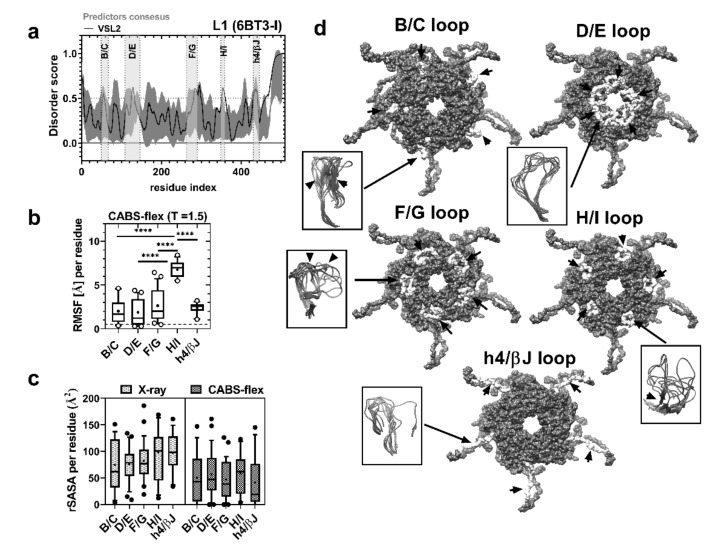
L1 epitopes are short disordered regions. (**a**) Prediction of the intrinsically disordered region of the L1 HPV16 sequence (6BT3-I). PONDR (black line) and predictor consensus (dark gray shades) are shown (dotted line, threshold = 0.5). The light gray boxes designate the described antigenic loops of the L1 viral protein (B/C, D/E, F/G, H/I, and h4/βJ). (**b**,**c**) RMSF (Å) from CABS-flex at T = 1.5 and average relative SASA (Å^2^) from L1 antigenic loops, evaluated by one-way ANOVA (**** *p* < 0.0001). (**d**) Representation of the surface accessibility of antigenic loops (white surfaces labeled with black arrows) in the L1 pentamer (PDB: 3J6R): the loops are representative of 10 models predicted by CABS-flex, and their ambiguous folding regions are indicated (black arrows).

Chimeric virus-like particles derived from the HPV16 major capsid protein L1 have been used as carriers of foreign epitopes to induce an immune response and antibody production [51,52,53]. A common strategy is to use insertion or substitution sites in the endogenous antigenic regions of the L1 protein to attach the epitope of interest based on information derived from B-cell epitope mapping [54,55]. Additionally, IDRs in viral capsid proteins have been used as targets for vaccine or drug therapies [56,57,58,59]. Thus, we used VLPs to evaluate the immunogenicity of sDE1 and sDE3. First, we analyzed the natural disordered region that was predicted from the experimental epitope map of the major capsid viral protein L1 (PDB: 6BT3). Figure 2a and Figure S1b show that the conserved and antigenic L1 loops from HPV are short IDRs: B/C (49–67), D/E (121–146), F/G (263–296), H/I (349–359), and h4/βJ (430–445) [52,53,54,55]. In addition, these five disordered loops of L1 have high flexibility scores: B/C = 2.021, D/E = 1.880, F/G = 2.647, H/I = 6.756, and h4/βJ = 2.395. However, the H/I loop proved to have more flexibility than the others (Figure 2b). Although the H/I loop has the highest flexibility of the loops, the accessibility to the solvent (rSASA average) is similar among all loops (Figure 2c). Analyses of the folding probabilities show high flexibility in the five loops with order–disorder transitions (beta-sheet formation), which is more evident for the B/C, F/G, and H/I loops (Figure 2d). These results demonstrate that the low-complexity structure (loop conformation) has a clear correlation with the antigenicity/binding site and with the intrinsic disorder (flexibility) of conserved L1 loops. These features might explain the efficiency of these epitopes in inducing high levels of cross-neutralizing antibodies against HPV-16, 31, 33, 35, 52, and 58 variants [60]. We selected the h4/βJ loop as a substitution site for carrying CRTAM sDE1 and sDE3 because it has been previously used as a carrier of the M2 influenza A protein, *Plasmodium falciparum* VAR2CSA protein, and amyloid-β peptides to raise specific antibodies [61,62,63].

### 2.4. Chimeric Constructs Were Assembled into Virus-Like Particles

**Figure 3:**
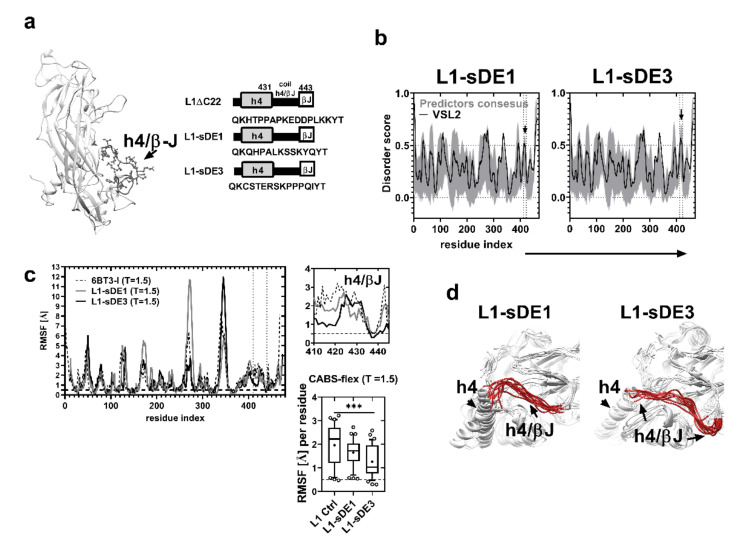
CRTAM sDEs retain their flexibility in chimeric L1 viral protein. (**a**) L1 3D model (the h4/βJ loop is indicated). Chimeric construct scheme (right) of each synthetic gene; different structures are indicated by gray shaded boxes (helix 4), white boxes (βJ), and thick black lines (h4/βJ loop). The top panel shows the native residue sequence (L1ΔC22), and the middle and bottom panels show the CRTAM epitopes that were replaced in the native sequence (L1-sDE1 and L1-sDE3). (**b**) Prediction of the intrinsically disordered region of chimeric viral protein sequences: the gray shaded areas and black line denote the predictor consensus and PONDR-VSL2, respectively; the residues that constitute IDR and CRTAM epitopes are indicated with black arrows. (**c**) Behavior of RMSF of chimeric viral proteins and control from CABS-flex MD at T = 1.5: the top panel (right) shows a close-up of a selected area corresponding to the h4/βJ loop (430–443); the bottom panel (right) shows the average RMSF of sDEs from chimeric viral proteins at T = 1.5, evaluated by one-way ANOVA (*** *p* < 0.001). (**d**) Three-dimensional models from chimeric L1 proteins: CRTAM sDEs in the h4/βJ loop are in red, and the h4 helix structures are indicated with black arrows.

**Figure 4:**
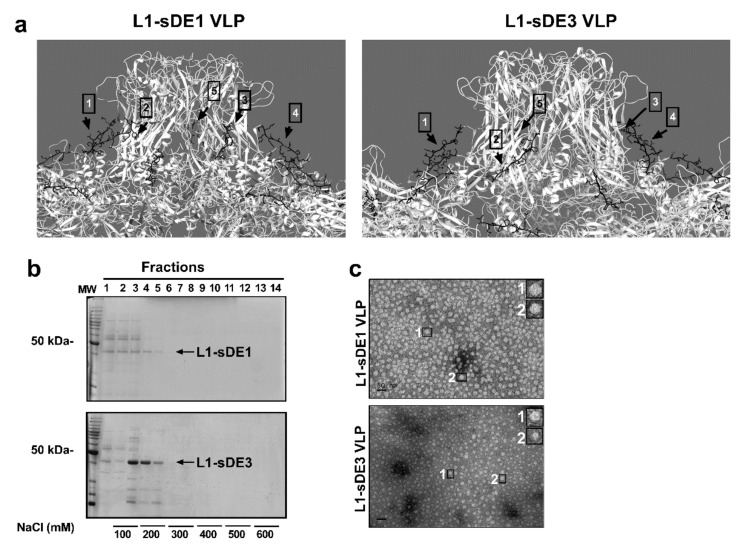
Chimeric viral proteins assembled into virus-like particles. (**a**) Full chimeric viral capsid of each construct reconstructed by crystal symmetry coordinates of the reported L1 structure: disordered epitopes are in black (sDE1 and sDE3), labeled with black arrows and numbers for each chimeric L1 monomer. (**b**) SDS-PAGE of the fraction from chromatographic purification of L1-sDE1 (top) or L1-sDE3 (bottom). Black arrows show the molecular weights of purified proteins, and the concentrations of NaCl used for purification are indicated at the bottom of the figure. (**c**) Transmission electron microscopy of chimeric viral proteins assembled into VLPs. A close-up view of assembled VLPs, scale bar = 50 nm.

To produce VLPs, we designed two synthetic genes (Figure 3a and Table S2) based on the L1ΔC22 protein reading frame with a plant codon-optimized sequence (GenBank: DQ067890.1) [64], here referred to as L1-sDE1 and L1-sDE3. These chimeric proteins were analyzed with the disorder predictors to evaluate whether they maintained their molecular disorder at the replacement site (430–445, h4/βJ loop). Figure 3b and Figure S1c,d show that both epitopes maintain their molecular disorder, which correlates with their flexibility score. Additionally, the CABS-flex analysis of loops at T = 1.5 reveals that RMSF does not differ between L1-sDE1 and the L1-crystal control, but there is a difference for L1-sDE3 (Figure 3c, bottom panel). This result agrees with the low flexibility of sDE3 observed in the Ig-like C1 model (Figure 1c,d). We tested the folding change that resulted from the loop substitution of proximal α-helix 4 (h4), which interacts with the neighboring h2 and h3 α-helices to form pentamers [65]. The results show that the folding structure of h4 in L1-sDE1 and L1-sDE3 is not affected; this indicates that the loop structure is well maintained, and its exposure to the solvent does not change (Figure 3d and Figure 4a, Figure S5b). Together, these data suggest that CRTAM disordered epitopes maintain their physical characteristics within the viral protein without disturbing the surrounding structures, which are important for the recruitment of capsomers.

Afterward, the chimeric viral proteins were produced in *Nicotiana benthamiana* by an agro-infection system. The purified viral proteins have a molecular weight of 45 kDa (fractions F4 and F5), as shown in Figure 4b. The TEM analysis shows that the chimeric capsomers are able to assemble into 40 nm VLPs (Figure 4c), as reported in other works [61,63,64,66].

### 2.5. Chimeric L1 Particles Induce Specific Antibodies That Recognize CRTAM

**Figure 5:**
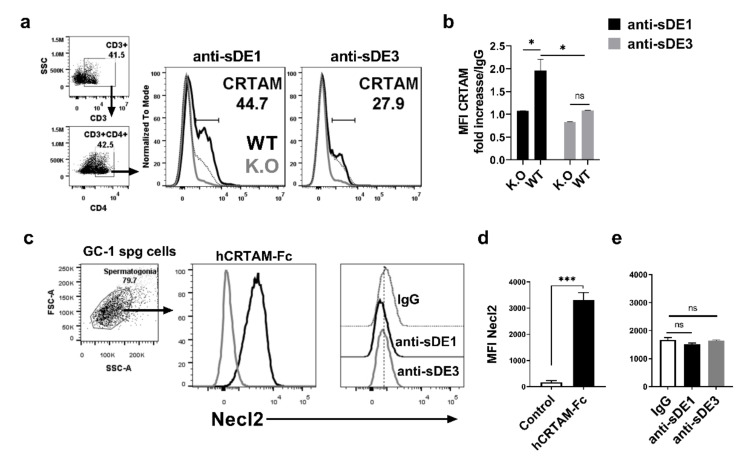
Polyclonal anti-sDE1 antibodies recognize the native CRTAM expressed in T cells and do not cross-react with Necl2. (**a**) Histogram of CRTAM expression in CD4 T cells from CRTAM knockouts (gray line) or from C57BL6 (black line). Cells were stained with polyclonal anti-sDE1, anti-sDE3, or polyclonal IgG (dotted line). (**b**) The bar graph displays the reactivity of polyclonal anti-sDE1 or anti-sDE3 normalized against the control (rabbit polyclonal IgG) as the mean fluorescence intensity (MFI) of CRTAM expressed in CD4 T cells. Splenocytes from C57Bl/6 mice or CRTAM knockout mice were stimulated with ionomycin/PMA for 14 h, and then cells were stained and analyzed by flow cytometry. Stimulated or unstimulated cells were gated on CD3^+^CD4^+^. The data are expressed as the mean and standard error from three independent experiments, with three wild-type mice and three KO mice in each experiment. (**c**) FACS and histogram of Necl2 expression in GC-1 spg cells. The spermatogonia cell line was stained with polyclonal anti-sDE1, anti-sDE3, or IgG. The soluble recombinant protein (hCRTAM-Fc) was used as a positive control, as previously reported [21,22,25,37]. (**d**,**e**) The bar graph shows the MFI of four conditions with three independent experiments. The statistical analysis was performed with one-way ANOVA, * *p* < 0.05, *** *p* < 0.001, ns = not significant.

To evaluate the antibody production of chimeric particles, we immunized New Zealand rabbits with 500 ug/mL of assembled VLPs and tested whether antisera recognized the hCRTAM-Fc recombinant protein. We observed that both antisera were able to recognize the CRTAM molecule, in contrast to pre-immune serum (Figure S8). These data confirm that both foreign epitopes were exposed on the surface of assembled chimeric VLPs and were able to stimulate the humoral and cellular response against the epitope, as reported for other antigens [67,68]. Further, we evaluated whether purified polyclonal antibodies (pAbs) against sDE1 and sDE3 could recognize the native folding of the CRTAM protein expressed in activated murine T cells. Splenocytes from C57BL/6 or from CRTAM KO mice were activated with ionomycin/PMA. The activated cells were stained with antisera and analyzed by flow cytometry. Anti-sDE1 identified a CRTAM^+^ population frequency of 44.7% (black line) in stimulated CD3^+^CD4^+^ cells from WT mice, which was not observed in stimulated CD3^+^CD4^+^ cells from CRTAM KO mice (gray line in Figure 5a, left panel). In contrast, the anti-sDE3 did not react with stimulated CD3^+^CD4^+^ T cells from WT mice (Figure 5a, right panel). These results are also supported by the fold increase in mean fluorescence intensity (MFI), calculated from the background of the IgG control (Figure 5b). However, both antisera reacted with the human recombinant hCRTAM-Fc in an ELISA assay, which demonstrates the presence of antibodies against the sDE3 epitope (Figure S8). This lack of reactivity of anti-sDE3 with the mouse activated CD3^+^CD4^+^T cells suggests that even though sDE3 is a linear epitope, it adopts a conformational orientation that is not accessible, thereby becoming a hidden epitope. However, the DE3 epitope is still exposed in the recombinant hCRTAM-Fc protein used in the ELISA test. Importantly, both sDE1 and sDE3 epitopes are conserved in mouse and human CRTAM (Figure S2 and Table S6).

We evaluated the cross-reactivity of both polyclonal antisera with Necl2. To achieve this goal, we used the GC-1 spg cell line, which has been reported to express high levels of Necl2 [69]. Necl2 expression was observed in GC-1 spg cells when hCRTAM-Fc recombinant protein was used as a positive control for the detection of this protein (Figure 5c,d). In contrast, no reactivity was observed with anti-sDE1 or anti-sDE (Figure 5e). This result reveals that despite some similarities among the extracellular domains between CRTAM and Necl2, sDE1 and sDE3 are specific to CRTAM.

## 3. Material and Methods

### 3.1. Animal Models

All animals used in this work were cared for following the animal protocol 0078-14, approved by the Internal Committee for the Care and Use of Laboratory Animals (CICUAL). The C57BL/6 mice and New Zealand rabbits were provided by the Unit for Experimentation and Production of Laboratory Animals (UPEAL) of the Center for Research and Advanced Studies (CINVESTAV), México City, México. The mouse strain B6:129S5-Crtamtm1Lex/Mmucd (RRID: MMRRC_032231-UCD) was acquired from the Knockout Mouse Project (KOMP) at the University of California, Davis, and housed by UPEAL.

### 3.2. Cell Culture

Splenectomies were performed on 4–6-week-old C57BL/6 and B6:129S5-Crtamtm1Lex/Mmucd (CRTAM K.O) mice. The spleens were disaggregated in PBS (pH = 7.4) + 1% FBS, and collected cells were washed with PBS. Cell viability was determined using Trypan blue exclusion. The splenocytes of C57BL6 and CRTAM KO mice were cultivated in 6-well plates (5 × 10^6^ cells per well) in 3 mL of RPMI + 10% SFB at 37 °C with 5% CO_2_. To create stimulation conditions, the cell culture was supplemented with 1 ug/mL of ionomycin and 20 ng/mL of PMA. The cell cultures were washed with PBS (pH = 7.4) + 1% FBS at 14 h post-activation and harvested for measuring CRTAM expression by flow cytometry.

### 3.3. B-Cell Epitope and Disorder Predictions

To identify CRTAM epitopes, the FASTA sequences of isoform-1 of murine CRTAM (UniProtKB: Q149L7-1), HPV16 viral capsid L1 (UniProtKB: P03101), and chimeric L1-sDE1 and L1-sDE3 proteins (see Table S2) were submitted to and analyzed by the following servers: BepiPred v1.0 [70,71], Emini Surface Accessibility Prediction [72], Karplus and Schulz Flexibility Prediction [73], and Parker Hydrophilicity Prediction [74], which are available on the online server of the Immune Epitope Database and Analysis Resource (IEDB) (URLs of the algorithms are listed in Table S3). Short epitopes (5-11 residues) localized in the Ig-like domains with a score of ≥0.5 were selected. The disorder prediction was performed by submitting the sequences mentioned above to Predictor Of Naturally Disordered Regions Algorithms (PONDR) [75,76] with the VSL2 algorithm [75,76], IUPRED2A [77], PrDOS [78], DISOPRED3 [79], and DisEMBL [80] (see Table S3). A 10% false-positive (FP) rate was used in all analyses to increase the sensitivity of predictions, and segments with scores of ≥0.5 were designated as disordered regions. All results were plotted in GraphPad Prism v8.0.

### 3.4. Multiple Alignment of Sequences

The CRTAM amino acid sequences of mammalian taxa (see Table S4) were evaluated by multiple alignment in ClustalX v2.0, and conserved residues for each epitope were visualized in Jalview v2.11. We constructed isoforms and hypothetical sequences from the UniProtKB database.

### 3.5. Structural Modeling Prediction and Validation

The sequences of murine CRTAM (UniProtKB: Q149L7) Ig-like V (residues 17–111), Ig-like C1 (residues 119–208), and chimeric L1-sDE1 and L1-sDE3 (see Table A.2) were submitted to the RaptorX web server [81]. The crystal structures of human CRTAM Ig-like V (PDB: 4H5S, chain A), PD-L1 (PDB: 5JDR, chain A), and HPV16 L1 (PDB: 6BT3, chain I) were used as the template. The 3D models were refined with the Refin2 web tool of the GalaxyWEB server to avoid collisions between atoms in the structure [82] and evaluated with RAMPAGE (URLs of web tools are listed in Table A.3). The models with the highest percentage of favorable coordinates (≥95%) were used for equilibration and molecular dynamics. The names of the chains for all PDB models were added using an online script (see Table S3).

### 3.6. Molecular Dynamics and Relative SASA Analysis

We performed coarse-grained molecular dynamics (MD) in the CABS-flex 2.0 web server [80] (see Table S3) for 10 ns. PDB structures of control models (4H5S-A, 5JDR-A, 6BT3-I) and query models (murine CRTAM Ig-like V and C1 domains; L1-sDE1 and L1-sDE3) were submitted to the server with standard restraint modes for helices and sheets (SS2, min = 3.8 and max = 8.0), with 100 cycles between trajectory frames for the simulation. To evaluate the fluctuation of flexibility, we ran the system at two different temperatures: T = 1.0, which represents the native crystallization parameters, and T = 1.5, which was used to increase the dynamics of the system. The resulting models were analyzed in UCSF Chimera v1.12, and the root-mean-square deviation (RMSF) per residue was plotted. In parallel, we performed 10 ns MD simulations using GROMACS v2018. The structure of each model was solvated using the OPLS-AA/L force field and water TIP3P model. Equilibration conditions were 300 K and 1 bar of pressure for 100 ps. The trajectories, temperature, and pressure fluctuations were analyzed in GROMACS and plotted. We evaluated the relative solvent-accessible surface area (rSASA) for the control and query models with DSSP (see Table S3). The rSASA per residue from the disordered epitope after CABS-flex simulation (T = 1.5) was compared with the control models (X-ray structures). All data were plotted in GraphPad Prism, and the means were evaluated by one-way ANOVA tests.

### 3.7. Construction and Expression of the Chimeric L1 Gene

To build the chimeric L1 genes, the nucleotide sequences that encode the disordered epitope QHPALKSSKYQ (localized in Ig-like V domain) or CSTERSKPPPQ (localized in Ig-like C domain) of murine CRTAM (NC_000075.6) were synthesized as the open reading frame (ORF) of the L1 protein sequence optimized for *Nicotiana benthamiana* codon use (AAY79402.1), lacking the first 27 residues of the N-terminus and the last 22 of the C-terminus (L1ΔC22). In both cases, the sequence that encodes residues 430–445, corresponding to the h4/βJ loop of L1, was replaced by sequences that encode the CRTAM disordered epitopes, here referred to as L1-sDE1 and L1-sDE3, respectively. Both chimeric genes were synthesized by GenScript^®^ flanked by BsaI sites. Each construct was subcloned into the expression vector pICH31070 and transferred into *Agrobacterium tumefaciens* strain GV3101. Four-week-old plants of *Nicotiana benthamiana* were agro-infected with each recombinant strain mixed at an equimolal ratio together with a magnifection system, following a protocol described previously [63].

### 3.8. Extraction and Purification of Viral Protein

The foliar tissue was processed ten days after agro-infection, and protein was extracted as described previously [63]. The extracts were purified with affinity chromatography in a HiTrap Heparin HP column (GE Healthcare, 17-0407-03). The samples were re-collected with 10 column volumes of elution buffer (citrate-phosphate buffer and NaCl gradient from 0.1 to 1.0 M). Afterward, the samples were dialyzed in PBS buffer (pH = 7.4) and analyzed using 12% SDS-polyacrylamide gel electrophoresis (SDS-PAGE).

### 3.9. Characterization of Chimeric Virus-Like Particles (VLPs)

To determine the size and morphology of chimeric virus-like particles, each viral capsid was disassembled and reassembled, as described previously [63]. Then, each sample was negatively stained with 2% phosphotungstic acid and prepared for transmission electron microscopy in a JEM-ARM200F Transmission Electron Microscope.

### 3.10. Immunization with Chimeric VLPs

Five-week-old male New Zealand rabbits were immunized intramuscularly with 600 ug of assembled VLP-sDE1 or VLP-sDE3 dissolved in 1 mL of PBS (pH = 7.4) every 15 days. The first two doses were supplemented with 500 uL of TiterMax^®^ Gold Adjuvant (Sigma-Aldrich, St Louis, MO, USA). Pre-immune sera were collected on day 1, and subsequent samples were taken one week after immunization. All animal experiments were performed according to the Guide for Laboratory Animals and Official Mexican Norms (NOM-062-ZOO-1999) and approved by the Cinvestav Institutional Committee for Care and Use of Laboratory Animals (approval number 0078-14).

### 3.11. Polyclonal Antibodies and Recombinant Protein Purification

IgG antibodies were purified with a column HiTrap Protein G H.P. (G.E. Healthcare, 17-0405-01) according to the manufacturer’s instructions. The hCRTAM-Fc recombinant protein was purified from the supernatant of stable transfected Jurkat/hCRTAM-Fc cells, as described previously [80].

### 3.12. Flow Cytometry

The cells (10^6^) were incubated with human gamma-globulins for 15 min at 4 °C, followed by incubation with the antibody cocktail. After incubation, the cells were washed with PBS (pH = 7.4), fixed with 4% paraformaldehyde, and analyzed in a BD LSR Fortessa cell analyzer (Biosciences). Lymphocytes were gated based on their light-scattering properties; then, they were gated as CD3^+^CD4^+^ T cells. The results were analyzed with FlowJo software (v.10.6.1; Tree Star, Inc., Ashland, OR, USA).

The antibody cocktail used was as follows: anti-CD3-APC/Cy7 (eBioscience, San Diego, CA, USA); anti-CD8-PE and anti-CD4 PerCP/Cy5.5 (R&D Systems, MN, USA); and anti-CD69-PE (eBioscience, CA, USA). The primary antibodies purified rabbit polyclonal anti-L1-sDE1, anti-L1-sDE3, or rabbit IgG (as the control), and Alexa Fluor 488 goat anti-rabbit was used as a secondary antibody (Invitrogen, Eugene, OR, USA).

### 3.13. Statistical Analysis

All graphs and statistical analyses were performed in GraphPad Prism v8.01.

## 4. Conclusions

In conclusion, we determined that sDE1 is an immunogenic epitope that is conserved among mice, humans, and many other mammalian species. sDE1 induces specific antibodies that recognize the disordered loop structure located in the contact interface of the CRTAM–Necl2 interaction. Thus, sDE1 may be a potential target site for blocking antibodies and has potential immunotherapeutic applications to treat inflammatory diseases, such as arthritis, rheumatoid, and multiple sclerosis.

## Data Availability

The data that support the findings of this study are available from the authors upon reasonable request.

## Supplementary Materials

The following are available online at https://www.mdpi.com/1422-0067/21/22/8798/s1. Table S1. Protein sequences and crystal structures used for homology modeling. Table S2. Synthetic ORF of chimeric L1 construction used in this work. Table S3. Software list used for prediction and analysis. Table S4. Epitope selection by BepiPred v1.0. Table S5. Intrinsically disordered regions according to the VSL2 algorithm of the PONDR predictor.Table S6. Mammalian sequences used for alignment in the UniProtKB database. Figure S1. Analysis of intrinsically disordered regions of proteins. Figure S2. Intrinsically disordered regions are conserved in mammalian species. Figure S3. Ramachandran plot of the 3D models generated. Figure S4. sDE1, sDE2, and sDE3 are loops. Figure S5. Relative SASA of CRTAM and chimeric L1 models. Figure S6. Molecular dynamics simulation of three-dimensional models of CRTAM Ig-like domains. Figure S7. Molecular dynamics of chimeric viral proteins. Figure S8. Polyclonal anti-IDR1 and anti-IDR3 recognize the recombinant protein hCRTAM-Fc.
