# Supplementary material for: Short Disordered Epitope of CRTAM Ig-Like V Domain as a Potential Target for Blocking Antibodies
Source: Int J Mol Sci. 2020 Nov 20;21(22):8798. doi: 10.3390/ijms21228798 (PMC7699905; doi:10.3390/ijms21228798)
Supplement: Supplementary file 1 [file ijms-21-08798-s001.pdf]

Short disordered epitope of CRTAM Ig-like V domain as a potential target for blocking antibodies

Julio Angel Vázquez-Martínez[1,2,3], Miguel Angel Gómez-Lim[3], Edgar Morales-Ríos[4], Jorge Alberto Gonzalez-y-Merchand[1], Vianney Ortiz-Navarrete[2,*]

[1]Departamento de Microbiología, Escuela Nacional de Ciencias Biológicas, Instituto Politécnico Nacional, Unidad Profesional Lázaro Cárdenas, Prolongación de Carpio y Plan de Ayala s/n, Colonia Santo Tomás, Delegación Miguel Hidalgo, C.P. 11340, Ciudad de México, México.

[2]Departamento de Biomedicina Molecular, Centro de Investigación y Estudios Avanzados del Instituto Politécnico Nacional (CINVESTAV), Av. Instituto Politécnico Nacional No. 2508, Colonia San Pedro Zacatenco, Delegación Gustavo A. Madero, C.P. 07360, Ciudad de México, México.

[3]Departamento de Ingeniería Genética, Centro de Investigación y Estudios Avanzados del Instituto Politécnico Nacional (CINVESTAV), Km 9.6 Carretera Irapuato-León, C.P. 36821, Irapuato, Guanajuato, México.

[4]Departamento de Bioquímica, Centro de Investigación y Estudios Avanzados del Instituto Politécnico Nacional (CINVESTAV), Av. Instituto Politécnico Nacional No. 2508, Colonia San Pedro Zacatenco, Delegación Gustavo A. Madero, C.P. 07360, Ciudad de México, México.

Correspondence

Dr. Vianney Ortiz-Navarrete, Departamento de Biomedicina Molecular, Centro de Investigación y Estudios Avanzados del Instituto Politécnico Nacional (CINVESTAV), Av. Instituto Politécnico Nacional No. 2508, Colonia San Pedro Zacatenco, Delegación Gustavo A. Madero, C.P. 07360, Ciudad de México, México. vortiz@cinvestav.mx

Supplementary Materials

Table S1. Protein sequences and crystal structures used for homology modeling.

Table S2. Synthetic ORF of chimeric L1 construction used in this work.

Table S3. Software list used for prediction and analysis.

Table S4. Epitope selection by BepiPred v1.0.

Table S5. Intrinsically disordered regions according to the VSL2 algorithm of the PONDR predictor.

Table S6. Mammalian sequences used for alignment in the UniProtKB database.

Figure S1. Analysis of intrinsically disordered regions of proteins.

Figure S2. Intrinsically disordered regions are conserved in mammalian species.

Figure S3. Ramachandran plot of the 3D models generated.
Figure S4. sDE1, sDE2, and sDE3 are loops.

Figure S5. Relative SASA of CRTAM and chimeric L1 models.

Figure S6. Molecular dynamics simulation of three-dimensional models of CRTAM Ig-like domains.

Figure S7. Molecular dynamics of chimeric viral proteins.

Figure S8. Polyclonal anti-IDR1 and anti-IDR3 recognize the recombinant protein hCRTAM-Fc.

Table S1. Protein sequences and crystal structures used for homology modeling

Name	UniProtKB ID	PDB ID	Chain	Query	Crystal parameters
CRTAM_MOUSE	Q149L7	-	-		
CRTAM_HUMAN	O95727	4H5S	A	Ig-like V	1.70 Å, 277 K, pH 8.0
VL1_HPV16	P03101	6BT3	I	Chimeric L1	4.70 Å, -, -
PD1L1_HUMAN	Q9NZQ7	5JDR	A	Ig-like C1	2.70 Å, 295 K, -

Table S2. Synthetic ORF of chimeric L1 construction used in this work.

Name	Translated ORF Sequence	ID
HPV-16 L1	>AAY79402.1 HPV-16 L1 [synthetic construct]<br>MSLWLPSEATVYLPPVPVSKVVSTDEYVARTNIYYHAGTSRLLAVGHPYFPIKKPNNNKILV<br>PKVSGLQYRVFRIHLPDPNKFGFPDTSFYNPDTQRLVWACVGVEVGRGQPLGVGISGHPLL<br>NKLDDTENASAYAANAGVDNRECISMDYKQTQLCLIGCKPPIGEHWGKGSPCTNVAVNP<br>GDCPPLELINTVIQDGDMVDTGFGAMDFTTLQANKSEVPLDICTSICKYPDYIKMVSEPYGD<br>SLFFYLRREQMFVRHLFNRAGTVGENVPDDLYIKGSGSTANLASSNYFPTPSGSMVTSDAQIF<br>NKPYWLQRAQGHNNGICWGNQLFVTVVDTTRSTNMSLCAAISTSETTYKNTNFKEYLRHG<br>EEYDLQFIFQLCKITLTADVMTYIHSMNSTILEDWNFGLQPPPGGTLEDTYRFVTSQAIACQK<br>HTPPAPKEDPLKKYTFWEVNLKEKFSADLDQFPLGRKFLLQAGLKAKPKFTLGKRKATPT<br>TSSTSTTAKRKKRKL	[1]
L1-sDE1	>L1-sDE1<br>MSLWLPSEATVYLPPVPVSKVVSTDEYVARTNIYYHAGTSRLLAVGHPYFPIKKPNNNKILV<br>PKVSGLQYRVFRIHLPDPNKFGFPDTSFYNPDTQRLVWACVGVEVGRGQPLGVGISGHPLL<br>NKLDDTENASAYAANAGVDNRECISMDYKQTQLCLIGCKPPIGEHWGKGSPCTNVAVNP<br>GDCPPLELINTVIQDGDMVDTGFGAMDFTTLQANKSEVPLDICTSICKYPDYIKMVSEPYGD<br>SLFFYLRREQMFVRHLFNRAGAVGENVPDDLYIKGSGSTANLASSNYFPTPSGSMVTSDAQI<br>FNKPYWLQRAQGHNNGICWGNQLFVTVVDTTRSTNMSLCAAISTSETTYKNTNFKEYLRH<br>GEEYDLQFIFQLCKITLTADVMTYIHSMNSTILEDWNFGLQPPPGGTLEDTYRFVTSQAIACQ<br>K**CSTERSKPPPQI**YTFWEVNLKEKFSADLDQFPLGRKFLLQAGLKAKPKFT<br>LG	608183-2<br>GenScript
L1-SDE3	>L1_sDE3<br>MSLWLPSEATVYLPPVPVSKVVSTDEYVARTNIYYHAGTSRLLAVGHPYFPIKKPNNNKILV<br>PKVSGLQYRVFRIHLPDPNKFGFPDTSFYNPDTQRLVWACVGVEVGRGQPLGVGISGHPLL<br>NKLDDTENASAYAANAGVDNRECISMDYKQTQLCLIGCKPPIGEHWGKGSPCTNVAVNP<br>GDCPPLELINTVIQDGDMVDTGFGAMDFTTLQANKSEVPLDICTSICKYPDYIKMVSEPYGD<br>SLFFYLRREQMFVRHLFNRAGAVGENVPDDLYIKGSGSTANLASSNYFPTPSGSMVTSDAQI<br>FNKPYWLQRAQGHNNGICWGNQLFVTVVDTTRSTNMSLCAAISTSETTYKNTNFKEYLRH<br>GEEYDLQFIFQLCKITLTADVMTYIHSMNSTILEDWNFGLQPPPGGTLEDTYRFVTSQAIACQ<br>K**QHPALKSSKYQ**YTFWEVNLKEKFSADLDQFPLGRKFLLQAGLKAKPKFTL<br>G	608183-3<br>GenScript

Table S3. Software list used for prediction and analysis.

Software	URL	Function
**IUPRED2A**	https://iupred2a.elte.hu/	Disorder
**PrDOS**	http://prdos.hgc.jp/cgi-bin/top.cgi	
**DisEMBL**	http://dis.embl.de/cgiDict.py	
**PONDR**	http://www.pondr.com/	
**DISOPRED3**	http://bioinf.cs.ucl.ac.uk/psipred/	
**CABS-flex 2.0**	http://biocomp.chem.uw.edu.pl/CABSflex2	MD
**GROMACS**	Free software v2018 (Linux system)	MD
**UCSF Chimera**	Free software v1.4 (Windows 64bits)	Structure analysis
**DSSP**	https://www3.cmbi.umcn.nl/xssp/	rSASA
**RaptorX**	http://raptorx.uchicago.edu/	S. Structure
**Refine2**	http://galaxy.seoklab.org/cgi-bin/submit.cgi?type=REFINE2	Refinement
**RAMPAGE**	http://mordred.bioc.cam.ac.uk/~rapper/rampage.php	Ramachandran Plot
**Rename Chain**	http://www.canoz.com/sdh/renamepdbchain.pl	Rename chain
**WebLogo**	https://weblogo.berkeley.edu/logo.cgi	Logo sequences
**Jalview**	Free software v2.11.0 (Windows 64bits)	Multi-alignment
**ClustalX**	Free software v2.1 (Windows 64bits)	Multi-alignment

Table S4. Epitope selection by BepiPred v1.0 (Threshold = 0.2).

Sequence	Position	Length (aa)	Label
VTVEEG	24-29	6	E1
SQTKN	39-43	5	E2
PALKS	62-66	5	E3
QNGEK	129-133	5	E4
TERSKPPPQI	141-150	10	E5
HEFEADGKIC	166-175	10	E6
YGKNS	186-190	5	E7
VADQETSDQETSDAPEQSSLSSQALQQPTSTVSMM	216-250	35	E8
NSSIPETDKEEKEHATQDPGLSTEASAQHTGLAR	252-285	34	E9
ESEISEQALESYRSRSNNEETSSQENSSQAPQSK	324-357	34	E10
SGAKTKKSAQHWKLGGKHSRVPES	368-391	24	E11

Table S5. Intrinsically disordered regions according to the VSL2 algorithm of the PONDR predictor.

Label	Sequence	Position	Score
**IDR1**	LKSSKYQL	65-71	0.5968
**IDR2**	NGEKSV	130-135	0.5304
**IDR3**	CSTERSKPP	138-147	0.586
**IDR4**	LVADQETSDQETSDAP EQSSLSSQALQQPTSTVSMMENSSIPETDK EEKEHATQDPGLSTEASAQHTGL	215-283	0.8673
**IDR5**	LESYRSRSNNEETSSQENSSQAPQSKRCMNYITRLYSGAKTKKSAQHW KLGGKHSRVPESIV	323-393	0.7555

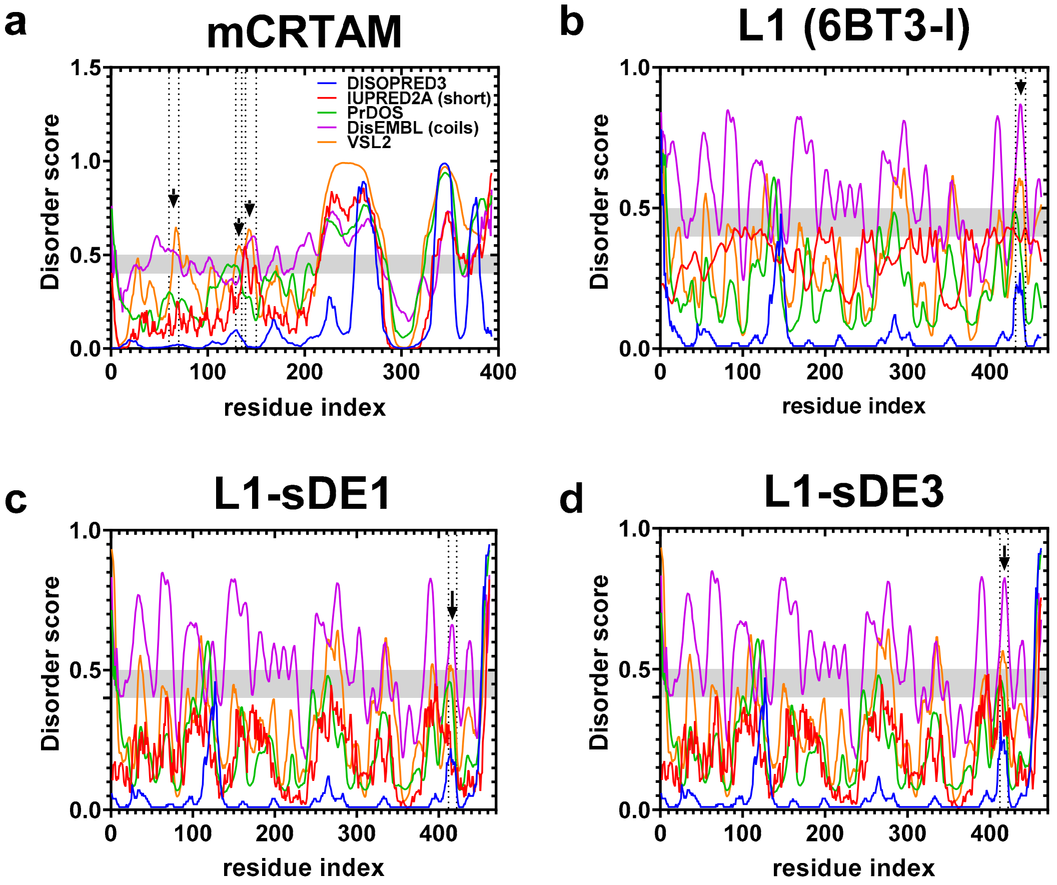

Figure S1. Analysis of intrinsically disordered regions of proteins. Result of murine CRTAM, major viral capsid HPV-16 L1, L1-sDE1, and L1-sDE3 proteins by DISOPRED3, IUPRED2A short, PrDOS, DisEMBL, and VSL2 predictors. A false-positive rate of 10% (FP = 10%) and a threshold rate of 0.4-0.5 (gray box) were used. The black arrows represent the position of disordered epitopes in the residue sequence. The X-axis is the residue position, and the Y-axis is the score of disorder.

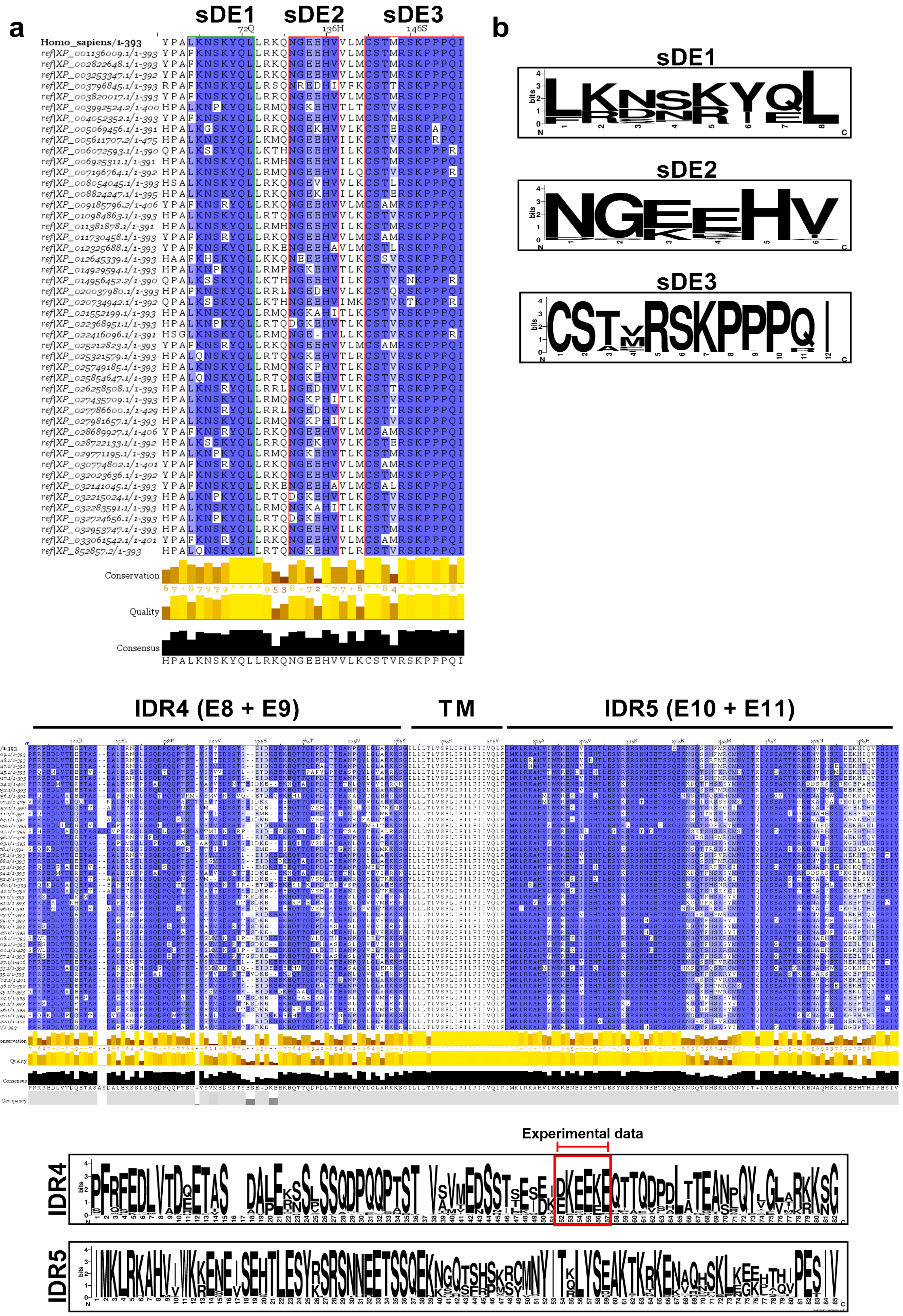
a
sDE1
sDE2
sDE3
Homo_sapiens/1-393
Conservation
Quality
Consensus
HPALKNSKYQLLRKQNGEEHVVLKCSTVRSKPPPQI
b
sDE1
sDE2
sDE3
c
IDR4 (E8 + E9)
TM
IDR5 (E10 + E11)
Conservation
Quality
Consensus
Occupancy
d
Experimental data
IDR4
IDR5

Figure S2. Intrinsically disordered regions are conserved in mammalian species. (**a**,**c**) Multiple alignment of the CRTAM sequence of mammalian species. Labels of epitopes found by BepiPred: short disordered epitopes (sDE1, sDE2, and sDE3) and long disordered region IDR4 (epitopes E8 + E9) or IDR5 (epitopes E10 + E11). (**b**,**d**) Sequence logos (frequency of residues in epitope) from the multiple alignment of mammalian taxa by the WebLogo tool. The short disordered epitopes are outlined by a green box (Ig-like V domain) or red box (Ig-like C1); blue shaded letters represent the conserved residues of the CRTAM protein between species; the colors of the bar graph represent the scores of the conservation and quality of sequences, from high scores in yellow columns to low scores in brown columns. The sequences were obtained by the UniProt database, aligned in ClustalX, and visualized in Jalview software. The experimental data of antibody production from the peptide DKEEKE reported previously [2], localized in the long IDR4, are outlined by the red box.

Table S6. Mammalian sequences from the UniProtKB database used for alignment.

Identifier	UniProtKB ID	Organism	Common Name
XP_001136009.1	H2R4X2	*Pan troglodytes*	*Chimpanzee common*
XP_002822648.1	H2NFM6	*Pongo abelii*	*Sumatra Orangutan*
XP_003253347.1	G1R764	*Nomascus leucogenys*	*Gibbon or northern white-cheeked gibbon*
XP_003796845.1	H0WRC0	*Otolemur garnettii*	*Northern greater galago*
XP_003820017.1	A0A2R9C440	*Pan paniscus*	*Bobono or pygmy chimpanzee*
XP_003992524.2	M3VW13	*Felis catus*	*Cat*
XP_004052352.1	G3RP88	*Gorilla gorilla gorilla*	*Western lowland gorilla*
XP_005069456.1	A0A1U7Q569	*Mesocricetus auratus*	*Golden hamster*
XP_005611707.2	F7CQM3	*Equus caballus*	*Horse*
XP_006072593.1	UPI00042CF7B5	*Bubalus bubalis*	*Water buffalo*
XP_006925311.1	L5JRN2	*Pteropus alecto*	*Black flying fox*
XP_007196764.1	A0A384BBU5	*Balaenoptera acutorostrata scammoni*	*minke whale*
XP_008054045.1	A0A1U7T8C8	*Tarsius syrichta*	*Philippine tarsier*
XP_008824247.1	UPI0004ED2004	*Nannospalax galili*	*Upper Galilee Mountains blind mole-rat*
XP_009185796.2	UPI0012AE19F9	*Papio anubis*	*Olive baboon*
XP_010984863.1	UPI00057BAD39	*Camelus dromedarius*	*Dromedary*
XP_011381878.1	UPI0005BA8E9D	*Pteropus vampyrus*	*Large flying fox*
XP_011730458.1	A0A2K6B282	*Macaca nemestrina*	*Southern pig-tailed macaque*
XP_012325688.1	A0A2K5EPX5	*Aotus nancymaae*	*Nancy Ma's night monkey*
XP_012645339.1	UPI000642E89E	*Microcebus murinus*	*Gray mouse lemur*
XP_014929594.1	UPI00072E9563	*Acinonyx jubatus*	*Cheetah*
XP_014956452.2	W5QBH9	*Ovis aries*	*Sheep*
XP_020037980.1	UPI00098167F6	*Castor canadensis*	*North American beaver*
XP_020734942.1	UPI000A1BC50A	*Odocoileus virginianus texanus*	*White-tailed deer*
XP_021552199.1	A0A2Y9HR62	*Neomonachus schauinslandi*	*Hawaiian monk seal*
XP_022368951.1	A0A2Y9K6F9	*Enhydra lutris kenyoni*	*Sea otter*
XP_022416096.1	A0A2Y9M4V2	*Delphinapterus leucas*	*Beluga whale*
XP_025212823.1	UPI000DC1B0E9	*Theropithecus gelada*	*Gelada*
XP_025321579.1	UPI000DC690E7	*Canis lupus dingo*	*Dingo*
XP_025749185.1	A0A3Q7QRJ6	*Callorhinus ursinus*	*Northern fur seal*
XP_025854647.1	A0A3Q7T3T2	*Vulpes vulpes*	*Red fox*
XP_026258508.1	UPI000E55F2A8	*Urocitellus parryii*	*Arctic ground squirrel*
XP_027435709.1	UPI000F7FBCBB	*Zalophus californianus*	*California sea lion*
XP_027786600.1	UPI000FFFA444	*Marmota flaviventris*	*Yellow-bellied marmot*
XP_027981657.1	UPI001016DC6B	*Eumetopias jubatus*	*Steller sea lion*
XP_028689927.1	UPI0010A293CC	*Macaca mulatta*	*Rhesus macaque*
XP_028722133.1	UPI0010A15881	*Peromyscus leucopus*	*White-footed mouse*
XP_029771195.1	UPI00115667F4	*Suricata suricatta*	*Meerkat*
XP_030774802.1	UPI0012375BA4	*Rhinopithecus roxellana*	*Golden snub-nosed monkey*
XP_032023636.1	UPI001362C943	*Hylobates moloch*	*Silvery gibbon*

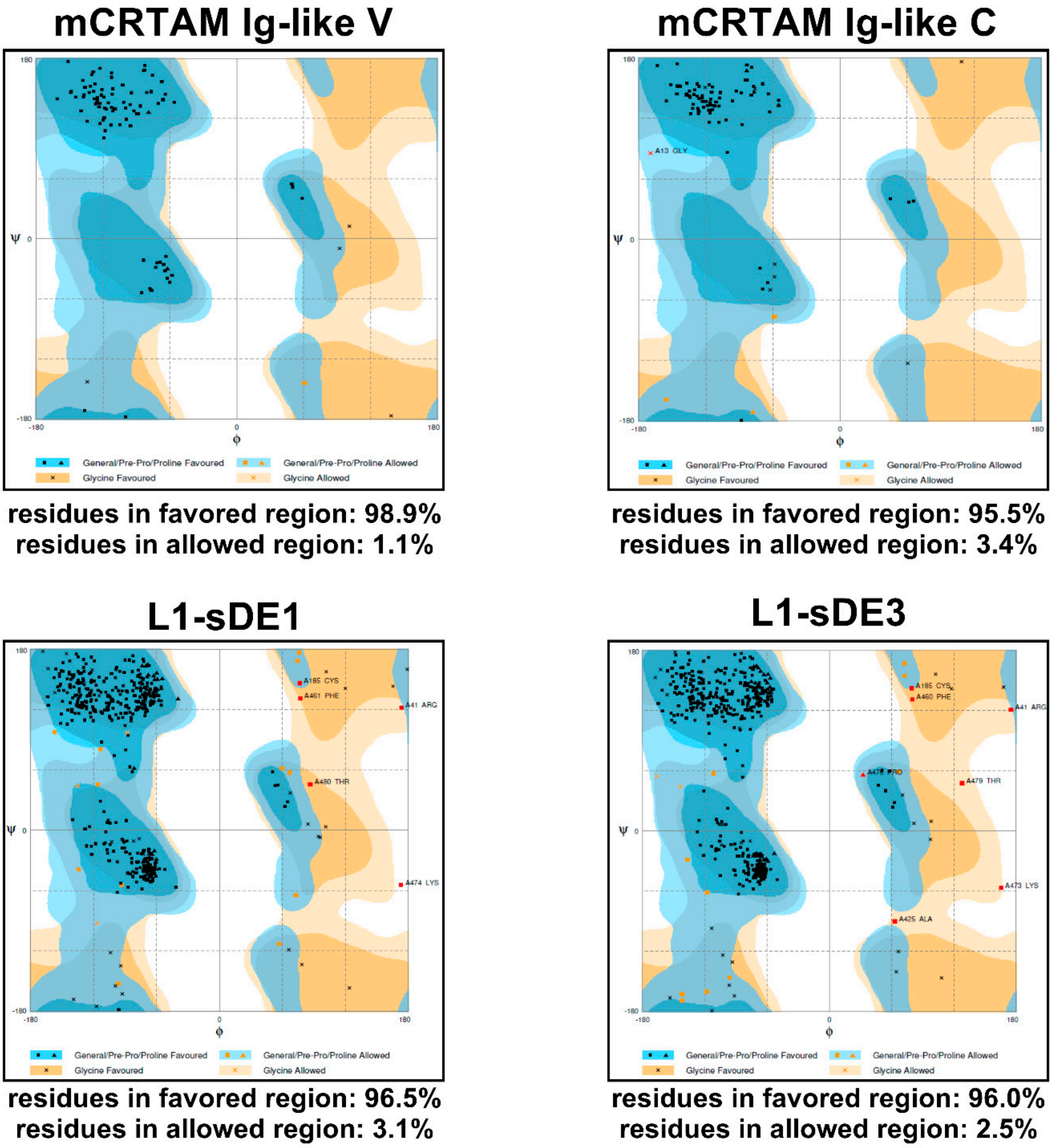

Figure S3. Ramachandran plot of the 3D models generated.

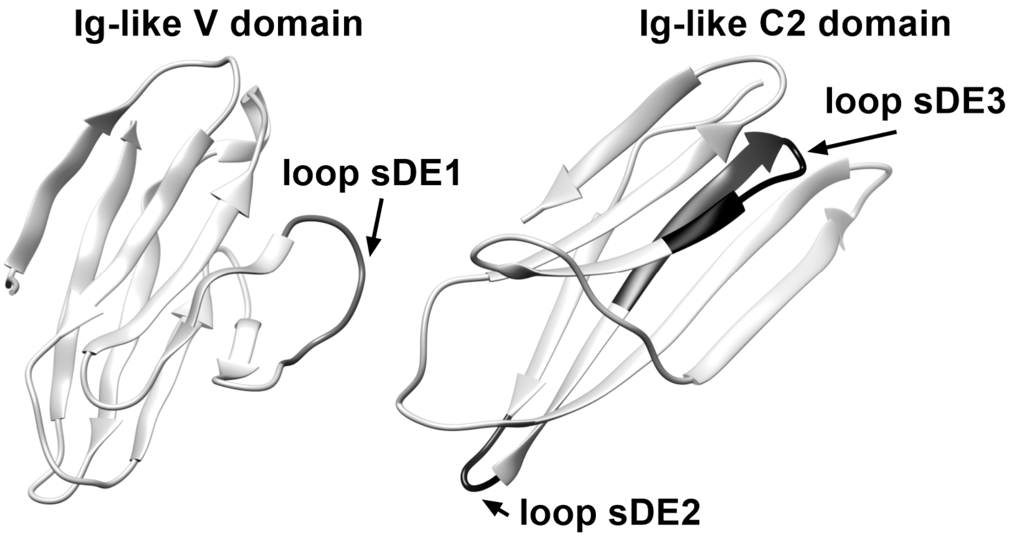

Figure S4. sDE1, sDE2, and sDE3 are loops. Models were generated in RaptorX and refined in the GalaxyWEB Refin2 tool. The loop structures of short disordered epitopes in murine CRTAM Ig-like domains are highlighted in black. The visualization was constructed in UCSF Chimera v1.4.

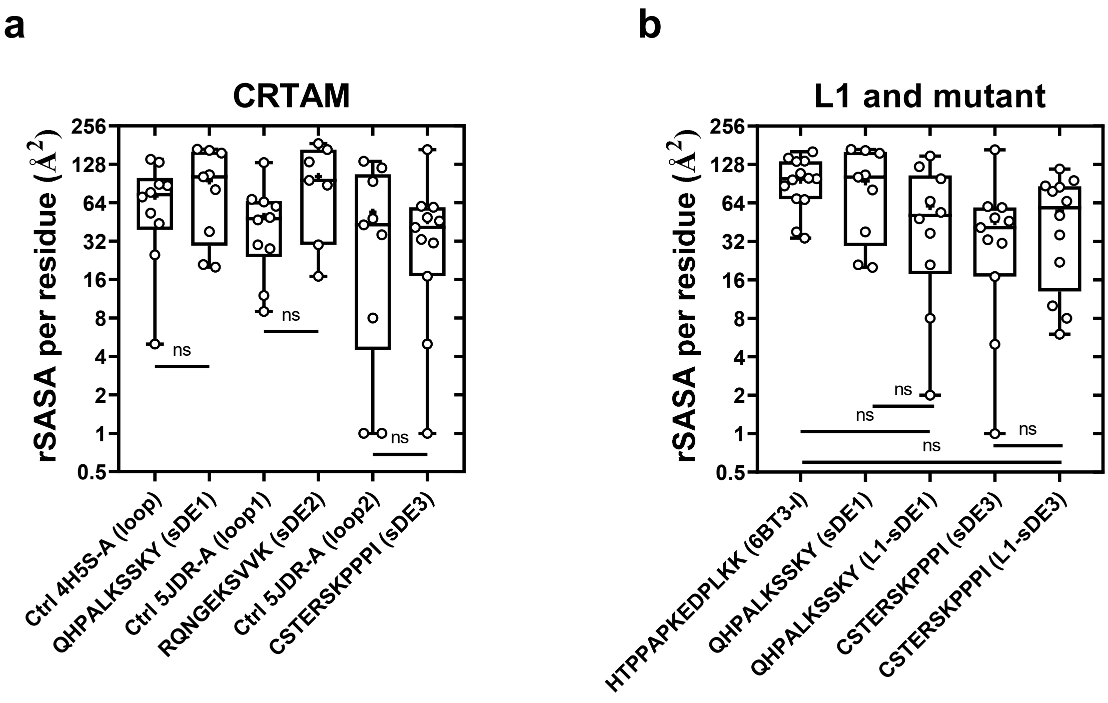

Figure S5. Relative SASA of CRTAM and chimeric L1 models. Values of the relative solvent-accessible surface area (rSASA) per residue, presented as percentile plots. The X-axis represents the experimental epitopes selected for the constructions; the Y-axis is the area in square Angstrom. Statistical analysis was performed as multiple comparisons with one-way ANOVA; *p = 0.1, **p = 0.001, ***p = 0.001, ns = not significant.

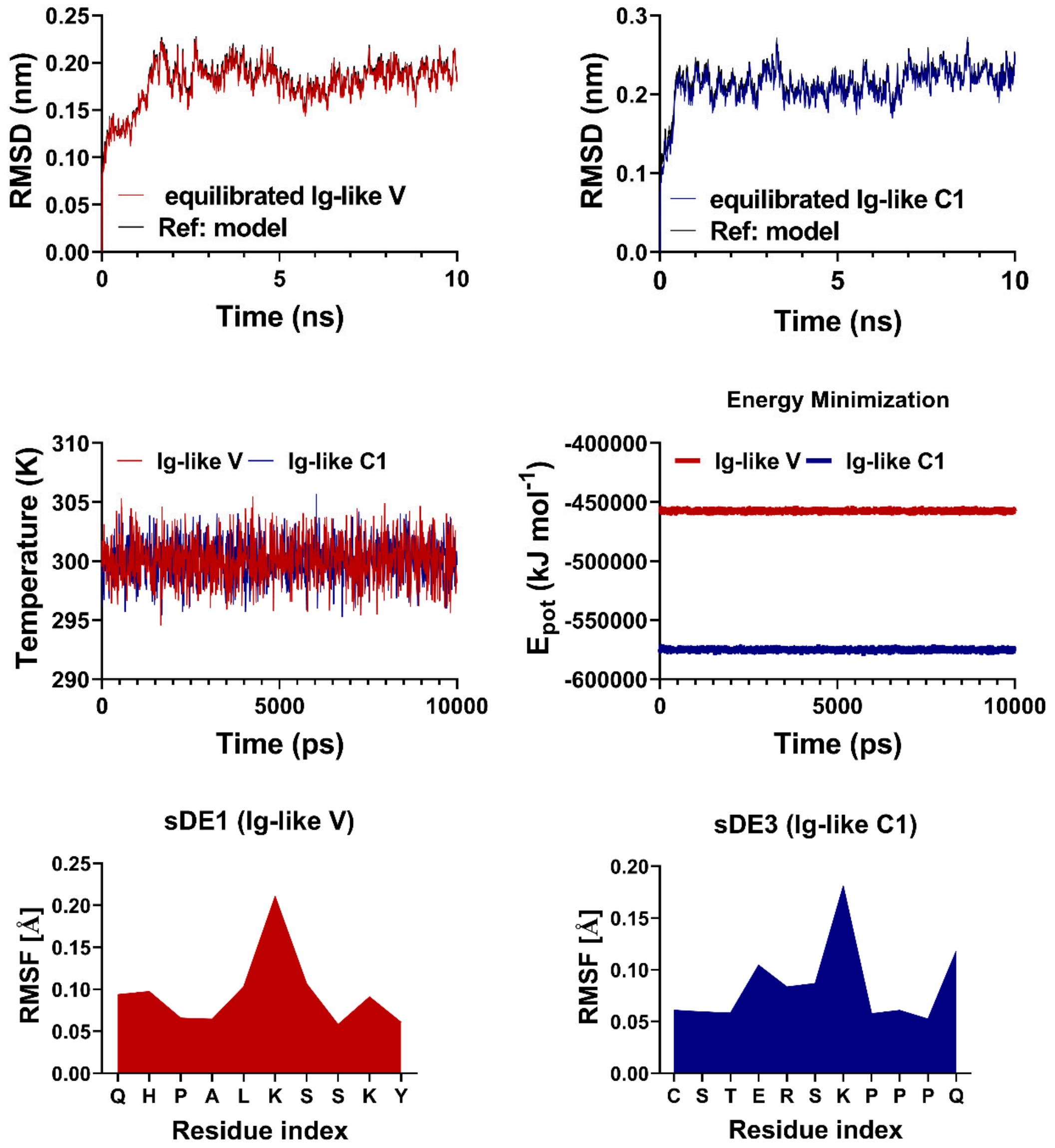

Figure S6. Molecular dynamics simulation of three-dimensional models of CRTAM Ig-like domains. Equilibrated three-dimensional Ig-like variable and constant C1 domains of the murine CRTAM protein after molecular dynamics. The potential energy, temperature, RMSD, and RMSF were evaluated for 10,000 ps.

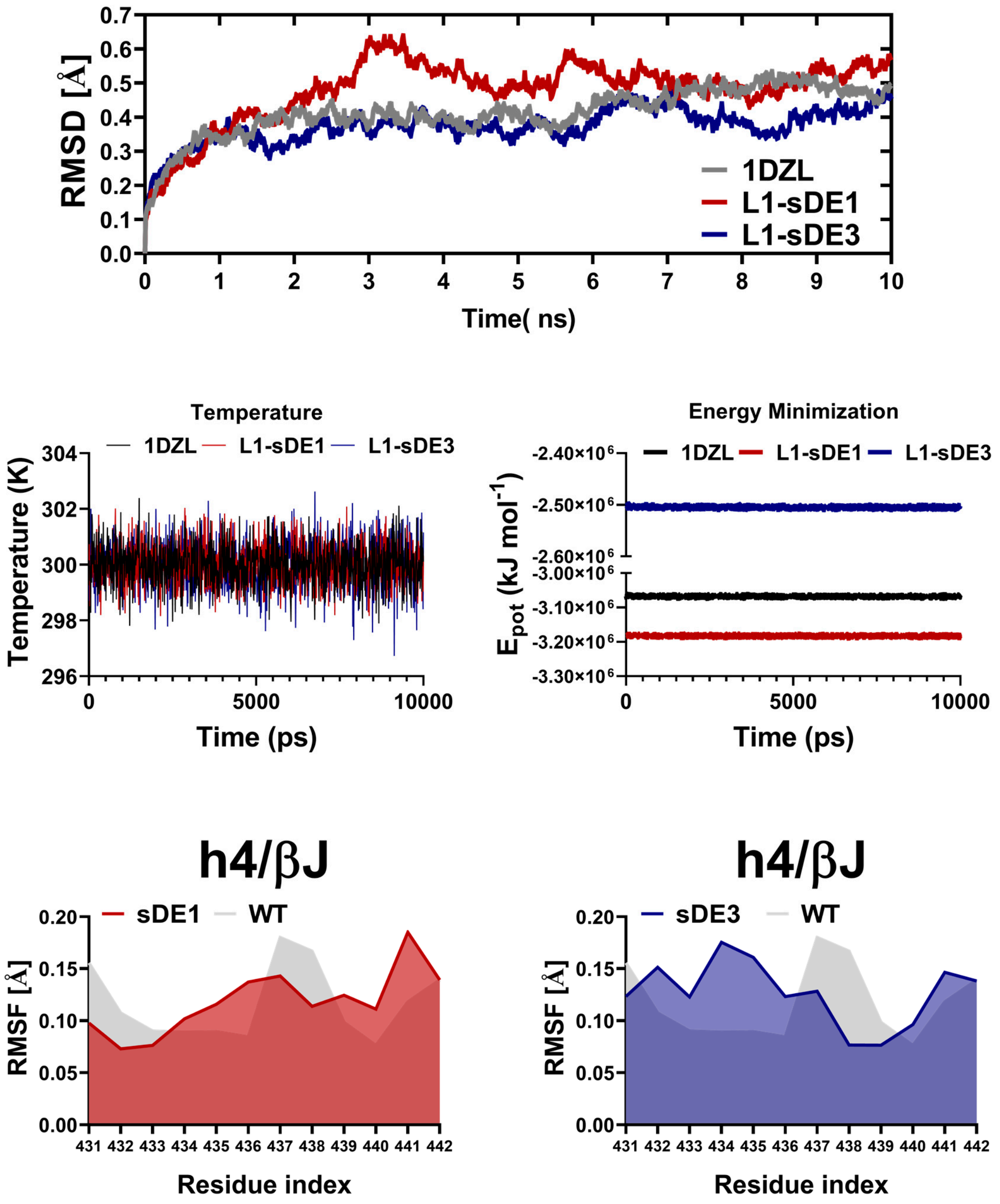

Figure S7. Molecular dynamics of chimeric viral proteins. Root-mean-square deviation of atomic positions (RMSD) and RMSF of the control model L1 (1DZL) and two chimeric viral proteins L1-sDE1/-sDE3 for 10,000 ps.

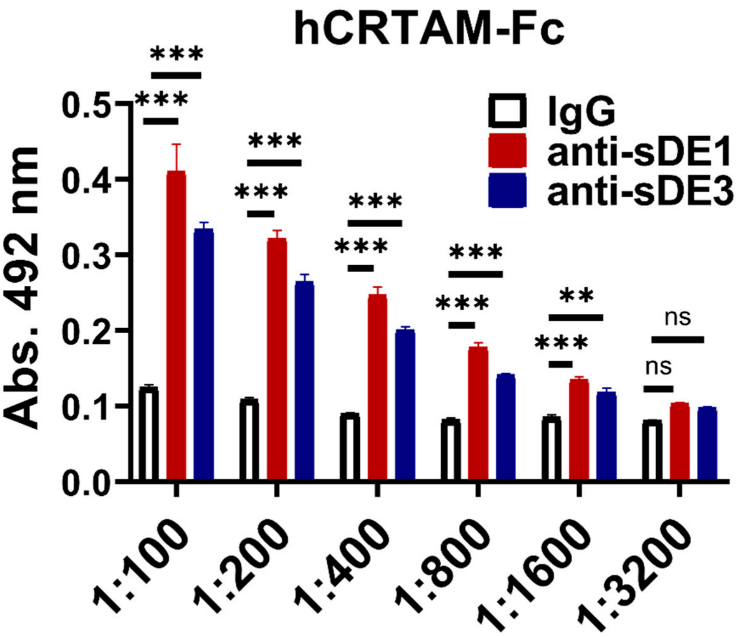

Figure S8. Polyclonal anti-IDR1 and anti-IDR3 recognize the recombinant protein hCRTAM-Fc. Representative bar graph of three independent ELISA assays. The data are expressed as the mean and standard error of duplicates from three independent experiments. Two-way ANOVA: *P < 0.05 **P < 0.01, ***P < 0.001, ns = not significant. ELISA was performed as follows. The plate wells were coated overnight with purified recombinant hCRTAM-Fc (extracellular region of human CRTAM fused to IgG1 Fc [2,3]) in carbonate buffer (pH 9.5) at 4 °C and blocked with 1X PBS supplemented with 10% FBS (1 h). Anti-sDE1, anti-sDE3, and purified rabbit IgG (used as a negative control for specificity) were diluted using a blocking buffer. Diluted antibodies were added to the plate wells and incubated for two hours at RT. After three washes, peroxidase-conjugated goat anti-rabbit IgG (CAT# AP132P, Sigma-Aldrich) diluted to 1:5000 in blocking buffer was added and incubated for 1 h at RT. After three washes, an o-phenylenediamine substrate solution (CAT# P8287, Sigma-Aldrich) was added, and after a 15-min incubation at 37 °C, the reaction was terminated with 2 N of H_2SO_4. Absorbance at 492 nm was read using an absorbance microplate reader (Tecan Sunrise, Salzburg, Austria).

Supplementary Reference

[1] X. Chen, T. Zhang, H. Liu, Y. Hao, G. Liao, X. Xu, Displaying 31RG-1 peptide on the surface of HPV16 L1 by use of a human papillomavirus chimeric virus-like particle induces cross-neutralizing antibody responses in mice, Human Vaccines & Immunotherapeutics. 14 (2018) 2025–2033. https://doi.org/10.1080/21645515.2018.1464355.

[2] E. Garay, G. Patiño-López, S. Islas, L. Alarcón, E. Canche-Pool, R. Valle-Rios, O. Medina-Contreras, G. Granados, B. Chávez-Munguía, E. Juaristi, V. Ortiz-Navarrete, L. González-Mariscal, CRTAM: A molecule involved in epithelial cell adhesion, Journal of Cellular Biochemistry. 111 (2010) 111–122. https://doi.org/10.1002/jcb.22673.

[3] O. Medina-Contreras, G. Soldevila, G. Patiño-Lopez, E. Canche-Pool, R. Valle-Rios, V. Ortiz-Navarrete, Role of CRTAM during mouse early T lymphocytes development., Developmental and Comparative Immunology. 34 (2010) 196–202. https://doi.org/10.1016/j.dci.2009.09.009.
